# Examining the role of stimulus complexity in item and associative memory

**DOI:** 10.3758/s13421-024-01590-z

**Published:** 2024-07-18

**Authors:** Ricarda Endemann, Siri-Maria Kamp

**Affiliations:** Department of Neurocognitive Psychology, Trier University, Johanniterufer 15, 54290 Trier, Germany

**Keywords:** Aging, Associative memory, Stimulus complexity, Late posterior negativity

## Abstract

Episodic memory comprises memory for individual information units (item memory) and for the connections among them (associative memory). In two experiments using an object pair learning task, we examined the effect of visual stimulus complexity on memory encoding and retrieval mechanisms and on item and associative memory performance. Subjects encoded pairs of black monochrome object images (low complexity, LC condition) or color photographs of objects (high complexity, HC condition) via interactive imagery, and subsequently item and associative recognition were tested. In Experiment 1, event-related potentials (ERPs) revealed an enhanced frontal N2 during encoding and an enhanced late posterior negativity (LPN) during item recognition in the HC condition, suggesting that memory traces containing visually more complex objects elicited a stronger effort in reconstructing the past episode. Item memory was consistently superior in the HC compared to the LC condition. Associative memory was either statistically unaffected by complexity (Experiment 1) or improved (Experiment 2) in the HC condition, speaking against a tradeoff between resources allocated to item versus associative memory, and hence contradicting results of some prior studies. In Experiment 2, in both young and older adults, both item and associative memory benefitted from stimulus complexity, such that the magnitude of the age-related associative deficit was not influenced by stimulus complexity. Together, these results suggest that if familiar objects are presented in a form that exhibits a higher visual complexity, which may support semantic processing, complexity can benefit both item and associative memory. Stimulus properties that enhance item memory can scaffold associative memory in this situation.

## Introduction

Memory for discrete information units (item memory) and for the connections between units that belong to the same experience (associative memory) are of great importance in our everyday life and are at the core of episodic memory. The present study examines the extent and manner in which perceptual complexity, as one aspect of the properties of the units that enter our memory system, affects memory encoding and retrieval mechanisms as well as item and associative memory performance.

Successful item memory requires encoding of the object along with its properties, allowing for a distinction of this item representation from other representations, a process that requires attentional resources (Ecker et al., 2007; Uncapher et al., 2006). Typically, stimuli that have more properties require additional resources for item-feature binding, more strongly involving higher cortical regions (Uncapher et al., 2006). Associative memory also requires cognitive resources allocated to the relevant encoding and retrieval processes, as associative memory is reduced under divided attention, and this reduction is sometimes reported to be larger than for item memory (e.g., Castel & Craik, 2003). Since both item and associative memory are resource-demanding, there may be a trade-off regarding the allocation of attentional resources on one type of memory versus the other. Indeed, Hockley & Cristi (1996) reported that when subjects are instructed to focus attention on item-specific information in an associative learning task, associative memory performance suffers and vice versa. Recording event-related potentials (ERPs) during encoding, Kamp and colleagues (2022) also reached the conclusion that a pronounced item-encoding focus may impair associative memory. In a group of older adults with below-median associative memory performance, an ERP effect indicative of perceptual item-encoding processes was observed in an associative memory task, which was absent in the high performers. Naveh-Benjamin and Kilb (2014) reported that young adults showed better item but poorer associative memory for visually degraded compared to non-degraded word pairs. Since visual degradation leads to increased effort and the recruitment of a more distributed network of brain regions to identify an item (Genetti et al., 2010), this is also consistent with a trade-off between resource allocation on item versus associative processing during encoding.

Partially dissociable neural processes are engaged in encoding and retrieval of item and associative memory (Davanchi & Wagner, 2002; Donaldson & Rugg, 1998; Kamp et al., 2017; Wilding & Rugg, 1996). However, in order for an item-to-item link to be established in the form of an associative memory representation, each item must be individually perceived and processed, which requires search processes (Treisman & Gelade, 1980; Wolfe, 1994) that analyze each item with its properties such as color and/or shape (perceptual level) and assign a linguistic meaning (semantic level) (Faubert, 2002; Zimmer & Ecker, 2010). Hence, item and associative memory likely do not operate completely independently of each other. In support of this view, some factors have similar effects on both aspects of episodic memory: For example, pictures are remembered better than words in both item (Maisto & Queen, 1992; Paivio & Csapo, 1973; Snodgrass & Asiaghi, 1977) and associative memory (Endemann & Kamp, 2022; Hockley, 2008; Hockley & Bancroft, 2011). Hence, some factors that lead to an attentional focus on, and/or an improvement in, item-encoding processing may simultaneously support associative memory, rather than leading to a trade-off.

In a recent study by Forester and Kamp (2023), subjects encoded sequentially presented object pairs with interactive imagery. In addition, upon presentation of the first item, in one condition subjects were asked to think of semantic associations regarding the item (semantic condition), in another condition they inspected its perceptual details (visual condition), and in the third condition there were no specific instructions (control condition). Behaviorally, higher item memory performance relative to lower associative memory performance was observed in both the semantic and visual conditions compared to the control condition, supporting the notion that a focus on item processing can lead to a trade-off with associative memory encoding. However, an analysis of the frontal slow wave, which indexes resource-demanding elaborative processing (Kamp & Zimmer, 2015), revealed a more differentiated pattern: In the semantic condition, an enhanced slow wave during item encoding was associated with both successful item and associative memory, whereas in the visual condition, an enhanced slow wave was associated with successful item but unsuccessful associative memory. This suggests that more in-depth semantic item encoding improves associative encoding on the individual trial level, while a focus on superficial and purely perceptual item-encoding processes tends to impair associative memory.

An open question is how the complexity of information units that enter our memory system influence memory-encoding processes and associative memory. Thus, some computational memory theories acknowledge item complexity as a relevant factor in encoding and retrieval processes (e.g., Shiffrin & Steyvers, 1997), but its role has thus far been scarcely studied empirically. Here, we consider an image as perceptually more complex if it exhibits enhanced variance in integral visual features like color, shape, or texture. Visually more complex stimuli presumably require a higher processing effort due to a larger number of features having to be processed (Faubert, 2002; Zimmer & Ecker, 2010). Further, visually complex images are more distinctive due to their richness of detail, which can lead to enhanced memorability (Saraee et al., 2020). Thus, complex stimuli may require additional attentional resources, on the other hand reducing resources available for processing associations to other objects, leading to a trade-off. However, following Forester et al. (2023), if stimulus complexity supports semantic processing, it may support both item and associative memory.

Testing the role of visual complexity in item and associative memory, Bender and colleagues (Bender et al., 2017) had participants study face-name pairs. The face images were either strongly standardized grayscale photos without background (low complexity (LC)) or color photos with different facial expressions, photographed from different angles, and including varied background features (high complexity (HC)). In young adults, memory for the face images benefitted from high complexity, but associative memory for the face-name association was unaffected by the manipulation. However, in older adults, enhanced item memory for complex stimuli was associated with reduced associative memory. This suggests that stimulus complexity, the processing of which presumably requires cognitive resources, may lead to a trade-off between enhanced item but reduced associative memory in some cases, such as in older adults who show reduced processing capacities (Bender et al., 2017). However, Bender et al.’s visually complex photos contained not only varied physical features of the faces, but also complex background information and variations in facial expressions (i.e., contextual information). As the authors themselves note, older adults’ reduced associative memory in the complex condition could have been due to an increased demand in binding item and contextual features within the images. Hence, the influence of the perceptual complexity of objects on item versus associative memory remains an open question.

### The present study

We examined the effect of the visual complexity of object images on memory encoding and retrieval processes, as well as on item and associative recognition performance. We presented participants with either black, schematic images (low complexity/LC) or colored naturalistic photos (high complexity/HC) of the same object types without a background in a pair learning task, followed by tests of item and associative recognition. In Experiment 1, we examined event-related potential (ERP) activity to gain insight into the neurocognitive processes of memory encoding, as well as item retrieval mechanisms that are affected by stimulus complexity on a neurocognitive level. Thereby, we lay the groundwork for an interpretation of potential mechanisms underlying the effects of visual complexity on item and associative memory performance (Experiments 1 and 2). In Experiment 2, we examined item and associative memory in young and older adults. Thus, older adults typically show a deficit in encoding and retrieving associations, while their item memory is relatively intact (Old & Naveh-Benjamin, 2008; Naveh-Benjamin, 2000). Older adults also exhibit reduced processing resources (Salthouse, 1996) and show a perceptual decline (Gulya et al., 2002; Park & Puglisi, 1985), which may lead to an additional demand in processing complex images. Hence, in Experiment 2 we examined whether stimulus complexity affects associative versus item memory differently in young versus older adults.

## Experiment 1

ERPs can provide insights into memory encoding (Mecklinger & Kamp, 2023) and retrieval (Rugg & Curran, 2007) processes beyond behavioral measures of memory performance, because they track neurocognitive (sub-) processes that are evoked during memory tasks and that may contribute to memory performance.

During encoding, relevant to the present study are early visual ERP components, the N400 and the (frontal) slow wave. Regarding early visual components, Bradley et al. (2007) found that photos of complex scenes elicited a larger frontal negativity, accompanied by a larger posterior positivity than less complex pictures (photos of objects with no background) in a time window of 150–250 ms. Similarly, Shigeto et al. (2011) reported that polygons with higher numbers of sides elicited a larger frontal N2, compared to less complex polygons. The frontal N2 is sensitive to both stimulus complexity and the attention-grabbing nature of stimuli (Folstein & Van Petten, 2008). Hence, to examine how stimulus complexity of object images affected early attentional processing, we focused on the frontal N2.

Second, the N400 is associated with semantic access, peaks between 300 and 500 ms, and has a centro-parietal distribution (Kutas & Federmeier, 2011). The N400 was thus analyzed to examine whether and how semantic access varied according to the visual complexity of object presentation. Finally, the frontal slow wave varies with working memory load or encoding effort and in the context of episodic memory tasks indexes of deep, elaborative, and associative processing (Mecklinger & Kamp, 2023). Prior research has shown that slow frontal activity during encoding is sensitive to strategic processes. For example, Lucas et al. (2017) reported that frontal activity starting 700 ms after the stimulus was modified by a concreteness manipulation within a pair-encoding task that supported the conceptual combination of the pair, but not in a control task. Hence, we analyzed frontal slow-wave activity to examine the role of strategic elaboration in the present task.

ERPs recorded during a recognition test can provide insight into the processes that are recruited to distinguish between previously studied (“old”) and not studied (“new”) stimuli. Based on the difference in ERP activity, referred to as the “old/new effect” (Friedman & Johnson, 2000), conclusions can be drawn about the properties of memory traces and the mechanisms by which they are retrieved. The "early mid-frontal old/new effect” is pronounced when familiarity, which is relatively fast and automatic, contributes to the retrieval of a study episode (Azimian-Faridani & Wilding, 2006; Woodruff et al., 2006). The "left parietal old/new effect" has a later onset (400–800 ms), is maximal at (left) parietal electrodes, and reflects recollection-based retrieval (Rugg et al., 1996; Rugg & Curran, 2007; Wilding & Rugg, 1996). Finally, the late posterior negativity (LPN) onsets around 700 ms and has a parieto-occipital scalp distribution (Mecklinger et al., 2016). The stimulus-locked LPN indexes a continued effortful search process targeted at a detailed reconstruction of the past experience with a stimulus, which extends to well after the recognition response (Mecklinger et al., 2016). The LPN is typically pronounced in associative (source) memory tasks, but it can also be elicited in item recognition when multiple features, such as color or shape, are retrieved (Cycowicz, 2001; Sommer et al., 2018).

We expected that complex images would elicit an enhanced frontal N2 during encoding, reflecting an early attention mechanism, and analyzed the N400 and the frontal slow wave to examine a potential modulation of semantic access and elaborative processing due to the stimulus complexity manipulation, respectively. Regarding the ERPs elicited in the item-recognition test, we expected an enhanced left parietal old/new effect in the HC, compared to the LC, condition, indicating that more and/or richer item features lead to enhanced recollection (Vilberg & Rugg, 2009). Furthermore, due to a larger number of features to be reactivated, a larger LPN was expected in the HC condition.

We expected higher item memory performance in the HC compared to the LC condition, because complex items have more diagnostic features for recognition. If associative memory benefits from rich encoding of multiple, natural visual features that are integral to the item, associative memory should also be enhanced in the HC condition. Alternatively, if complex stimuli lead to a prioritization of item over associative encoding, a relative impairment in associative memory should be observed.

### Methods

Both experiments, including all methods and procedures, were reviewed and approved in advance by the local ethics committee at the University of Trier.

Participants from Experiment 1 in the LC condition also served as the young adult comparison group for a recently published study (Kamp et al., 2022). In this prior article, we reported behavioral data and ERP subsequent memory effects recorded during encoding; the ERPs recorded during retrieval and for the HC condition have not been previously published. Note also that no older adults were assigned to the HC condition in Experiment 1. The present analysis contains only data from young adults.

#### Participants

At the core of the present design is a condition (HC vs. LC; between subjects) × memory type (item vs. associative memory; within-subjects) interaction of a 2 × 2 mixed ANOVA. Given a desired power of 1-β = 0.9, α = 0.05, in order to detect an expected medium effect size of f = 0.25, the required sample size is *N* = 46 (Faul et al., 2007). Forty-six young adults (38 females, eight males; 19–37 years old, M = 22.93 years, SD = 3.48) participated in exchange for either partial course credit or a monetary compensation. The participants assigned to the two conditions (n = 23 each) did not differ in age or gender (both *p*-values > .11).

#### Procedure and task

After signing an informed consent form, the EEG preparations took place, which took 15–45 min. Afterwards, the memory task began. The task was identical for both conditions, except for the stimulus material (see next section).

**Fig. 1:**
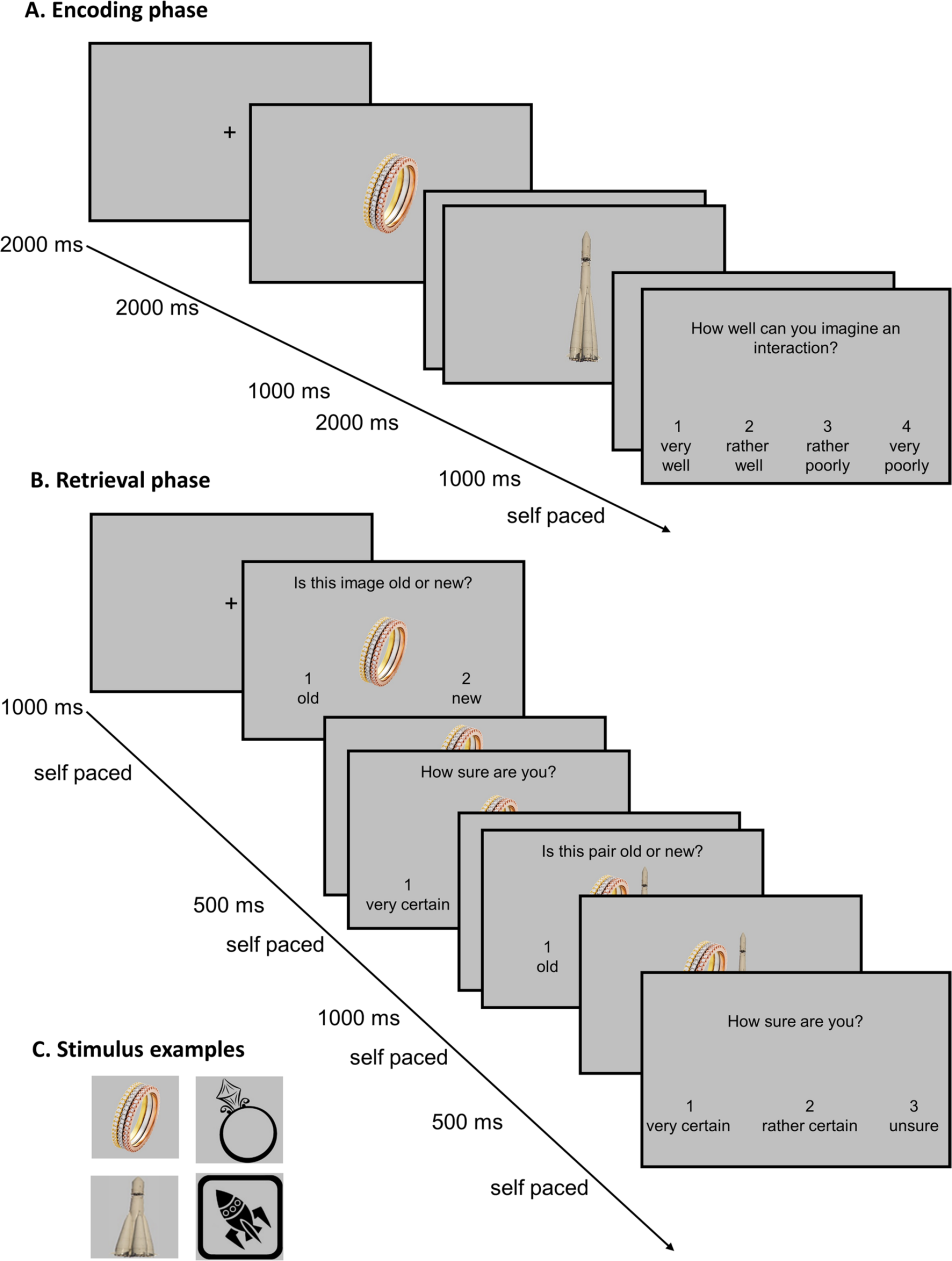
Trial structure in the encoding (**A**.) and recognition phase (**B**.). In the two-step recognition phase (B.), the associative test was completed only if the item, in this example the ring, was classified as old, regardless of the correctness of this answer, in experiment 1. In Experiment 2, the association test followed when the object was actually old, and not when the item was actually new, regardless of the participant’s answer. (C.) shows stimulus examples from the HC (left) and the LC condition (right)

E-prime 2.0 (Psychology Software Tools) was used to administer the task. Participants were instructed to encode pairs of unrelated objects via interactive imagery (i.e., the two objects should be mentally imagined in an interaction). The 120 object pairs were presented on a gray background in a sequential trial structure (Fig. 1): After a fixation cross (2,000 ms), an object was presented for 2,000 ms, followed by another fixation cross (1,000 ms) and the second object (2,000 ms) and another fixation cross (1,000 ms). Finally, participants were prompted to judge their ability to generate an interactive image on a scale of 1 (“very well”) to 4 (“very poorly”) on the computer keyboard. After the answer was given, a new trial started. The subject could take a break after every 40th trial.

After the encoding task, in a 5-min break the participants filled out questionnaires. Then, the recognition phase began. In the first part of a recognition test trial, the item test was conducted, in which one of 180 objects (120 old objects previously learned, which were always the first object of a pair, and 60 new objects not studied before) were presented. The item test began with a fixation cross (1,000 ms), followed by the object. Participants were asked to answer whether the object was old (“1”) or new (“2”). After the subject’s response, the object remained visible for 500 ms, and next the prompt for a confidence judgment was displayed, with gradations of “very certain” (1), “rather certain” (2), and “uncertain” (3). If the first object had been classified as “new” (2), the next trial began. The associative memory test followed only if the object had been rated as “old” (1). Proceeding to the associative test only for correct item-recognition judgments was done in order for the subject not to receive any feedback about the correctness of their response in the item test.

In the associative test, the second object could either be from the same pair as in the encoding phase (“old”; 2/3 of the trials) or from a different old pair ("recombined"; 1/3 of the trials). Both items of a study pair that was designated to be presented as recombined during test were utilized in different recombined trials. The study pairs that were presented as old versus recombined during the test were randomly selected. If a new object was falsely classified as "old," it was paired with another new distractor; associative memory performance was not evaluated in these trials. Each object was presented only once during the test. In the associative test, after a fixation cross (1,000 ms), two objects were presented simultaneously together with the question “Is this pair old or new?”, with the answer options of “old” (1) and “new” (2). After the response, the pair remained visible for 500 ms, and the confidence judgement was prompted. After every 45 trials, participants were allowed to take a break.

#### Stimuli

Stimuli for the LC condition included black line drawings and cliparts selected from the International Picture Naming Project (Szekely et al., 2004), Kamp and Donchin (2015), and an online search. The HC objects were drawn from internet searches and from different databases used in prior experiments of our research group. Care was taken to select pictures that displayed the same types of objects as in the LC condition, which we achieved for 266 out of 300 stimuli. For the remaining 34 stimuli, similar objects from the same categories were selected. The objects were from the categories “Household objects,” “Food,” “Vehicles,” “Musical Instruments,” “Clothing and Beauty,” “Humans,” “Sports and Recreation,” “Animals,” “Tools,” “Weather and Nature,” “Medicine,” “Furniture,” and “Buildings.” A full list of English object labels referring to the used stimuli is provided in the Appendix.

To confirm that our stimulus complexity manipulation was successful, for each image we extracted information on two stimulus aspects as proxies for complexity. First, we extracted the number of colors (including the gray levels) with a Python function that finds the best matches between detected color codes and source color codes (Shegocodes, 2022). Second, the number of edges was counted (after converting each image to grayscale) using cv2.Canny (OpenCV, 2023). Both the number of colors and edges were significantly larger for the HC (edges: M = 4829, SD = 3435; colors: M = 53.51, SD = 24.56) than the LC condition (edges: M = 4060, SD = 1880; colors: M = 14.62, SD = 1.95; both *p*-values < .01).

All objects were shown without background and were scaled to be of the same size. Individual objects were randomly assigned to object pairs in the encoding phase for each participant anew.

#### Analysis of behavioral data

All analyses were completed using JASP 0.16.4. To analyze the interactive imagery ratings from the encoding phase, we collapsed over 1 ("very well") and 2 ("rather well") ratings. A t-test for independent samples was calculated to compare this proportion of “well” ratings across conditions.

Corrected recognition scores (Pr-scores: hit rate – false alarm rate; Snodgrass & Corwin, 1988) were calculated separately for item and association memory and were analyzed in a mixed ANOVA with the between-subject factor condition (LC vs. HC) and the within-subject factor test type (item vs. associative). Note that due to the difference in the memory measure, main effects of test type are not of interest, but are nevertheless reported for the purpose of completeness.

In addition, we computed Bayesian ANOVAs to identify the most likely statistical model given the data and to compare the fit of competing models. We report BF_10_, which indicates the likelihood of a given model to explain the data, compared with the best model.

#### EEG recording and analysis

The EEG was recorded using a NeurOne Tesla amplifier (Bittium Cooperation, Finland) and the 10-20 system with 32 silver-silver chloride (Ag/AgCl) electrodes in the following positions: Fp1/2, F7/8, F3/4, Fz, FC5/6, FC1/2, T7/8, C3/4, Cz, CP5/6, CP1/2, TP9/10, P7/8, P3/4, Pz, PO9/10, O1/2, Iz. The electrode at the AFz position served as the ground electrode. The reference electrode was FCz, the data were off-line re-referenced to electrodes TP9 and TP10 (corresponding to the mastoid locations), and the signal at electrode FCz was reconstructed with the new reference. Analog-to-digital conversion was performed at a sampling rate of 500 Hz. Data were analyzed using Brain Vision Analyzer 2.1 (Brain Products GmbH).

The signal was filtered using an IIR Butterworth filter with a low cutoff frequency of 0.1 Hz and an upper cutoff frequency of 15 Hz. Segments time-locked to the first and second stimulus during encoding, and to the item-recognition test, were extracted from 400 ms before to 2,000 ms after the onset of the respective stimulus. Horizontal and vertical eye movements were corrected using the detection algorithm of a semi-automatic independent component analysis (ICA). Segments that contained remaining artifacts after the ICA were automatically removed if they exhibited an amplitude difference of 200 μV within a trial or an amplitude step of 30 µV/ms.

Artifact-free trials were averaged, and the averages were baseline-corrected. For the encoding phase, subject averages were calculated separately for the first and second stimulus and for trials for which the imageability was rated as high (1 or 2) versus low (3 and 4) (trial numbers: LC: stim 1/high: M = 77.13, SD = 20.60, stim 1/low: M = 41.52, SD = 20.55, stim 2/high: M = 77.43, SD = 20.61, stim 2/low: M = 41.30, SD = 20.52; HC: stim 1/high: M = 71.04, SD = 12.85 stim 1/low: M = 46.26, SD = 13.16, stim 2/high: M = 70.39, SD = 12.97, stim 2/low: M = 47.04, SD = 13.27). Subject averages for the item-recognition test included “old” or “new” trials (regardless of the participants’ response; trial numbers: LC: old: M = 97.70, SD = 28.13; new: M = 50.91, SD =11.15; HC: old: M = 103.74, SD =18.68; new: M = 52.04, SD =7.83).

We statistically analyzed the ERPs by means of mass univariate ANOVA with a cluster-based permutation test (Groppe et al., 2011; Fields & Kuperberg, 2020) in Matlab. This procedure allows for the identification of spatio-temporal clusters of significant ERP effects while controlling for the overall alpha-error of a given analysis (which was set to 0.05). The encoding ERP analysis included the within-subject factors stimulus (first vs. second) and imagery ability (high vs. low). The item recognition analysis included the within-subjects factor old/new. All analyses included the between-subjects factor condition (LC vs. HC). Spatio-temporal regions of interest focused on typical time windows and electrode regions for the respective components and are specified in the *Results* section.

### Results

#### Behavioral data

Behavioral data for both experiments are available via the Open Science Framework at https://osf.io/t7ayv/?view_only=af0365fb0ae1485bb535767deff0ab0f.

**Fig. 2:**
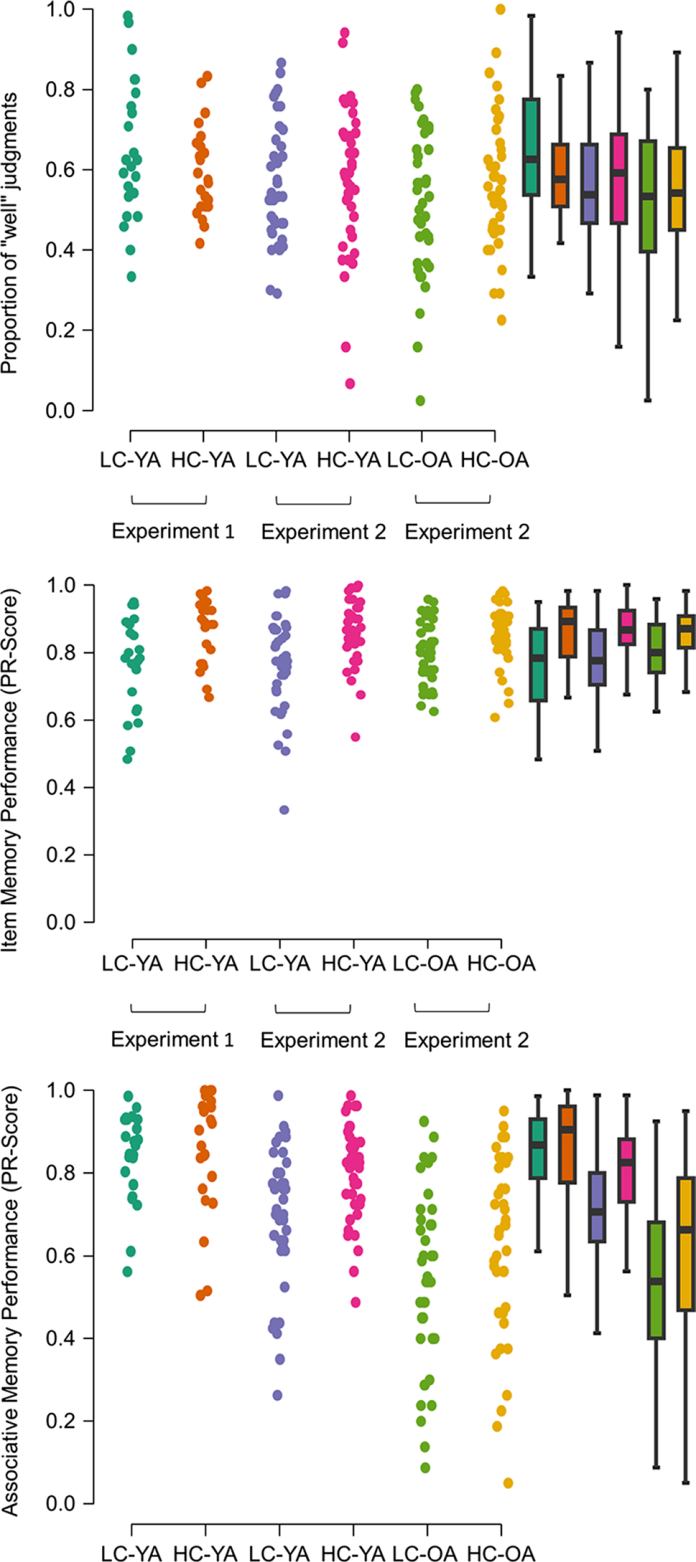
Behavioral data from experiments 1 and 2. Upper panel: Proportion of “well” judgments (“very well” and “rather well”) during study. Individual corrected recognition scores (hit rate-false alarm rate) for item (middle panel) and associative (bottom panel) recognition. Box plots are shown on the right of each panel. HC: high complexity, LC: low complexity, YA: young adults, OA: old adults

Although there was a higher tendency for participants in the LC condition to rate the interactions as easy to imagine (M = .65, SD = .18), compared to the HC condition (M = .60, SD = .11), the difference was not significant, *t*(44) = 1.24, *p* = .22 (Fig. 2). However, according to the Bayes factor of *BF*_*10*_* =* .55, there was only weak evidence for the null model against a model including a condition difference. The results hence remained inconclusive regarding a potential difference between conditions in study ratings.

Pr-scores by condition (LC vs. HC) and test type (item vs. associative) are illustrated in Fig. 2. The 2 × 2 ANOVA revealed a main effect of test type, *F*(1, 44) = 4.26, *p* < .05, η_p_^2^ = .09, and an interaction of test type × condition, *F*(1, 44) = 4.26, *p* = .005, η_p_^2^ = .16. The Pr-scores tended to be generally higher in the HC than in the LC condition, but this effect was not significant, *F*(1, 44) = 3.00, *p* = .09, η_p_^2^ = .06. The interaction indicated that the item Pr-scores were higher in the HC than in the LC condition, but the associative Pr-scores did not differ between conditions (Table 1, Fig. 2). The Bayesian model comparison indicated that the most likely model given the data contained both main effects and an interaction. The second-best fitting model contained two main effects but no interaction. The likelihood of this model was only about a tenth of the best model *BF*_*10*_ = .13, thus yielding moderate evidence for the model including the interaction.

**Table 1:** Memory performance: Means (+/- SD) for hit rates (HR), false alarm rates (FAR) and corrected recognition scores (PR) by experiment, condition, and test type

	Experiment 1				Experiment 2							
	Young adults				Young adults				Older adults			
	n = 46 (23/23)				n = 80 (40/40)				n = 80 (40/40)			
	Item		Associative		Item		Associative		Item		Associative	
	LC	HC	LC	HC	LC	HC	LC	HC	LC	HC	LC	HC
HR	.86 (.10)	.90 (.07)	.93 (.06)	.95 (.05)	.88 (.70)	.92 (.06)	.80 (.11)	.88 (.07)	.88 (.07)	.94 (.05)	.74 (.13)	.81 (.13)
FA	.09 (.04)	.04 (.04)	.09 (.09)	.09 (.13)	.11 (.10)	.08 (.15)	.11 (.09)	.08 (.08)	.08 (.06)	.08 (.09)	.21 (.17)	.20 (.21)
PR	.76 (.14)	.87 (.10)	.84 (.11)	.85 (.15)	.77 (.14)	.84 (.06)	.70 (.17)	.80 (.12)	.80 (.09)	.85 (.09)	.54 (.21)	.60 (.24)

#### ERPs at encoding

**Fig. 3:**
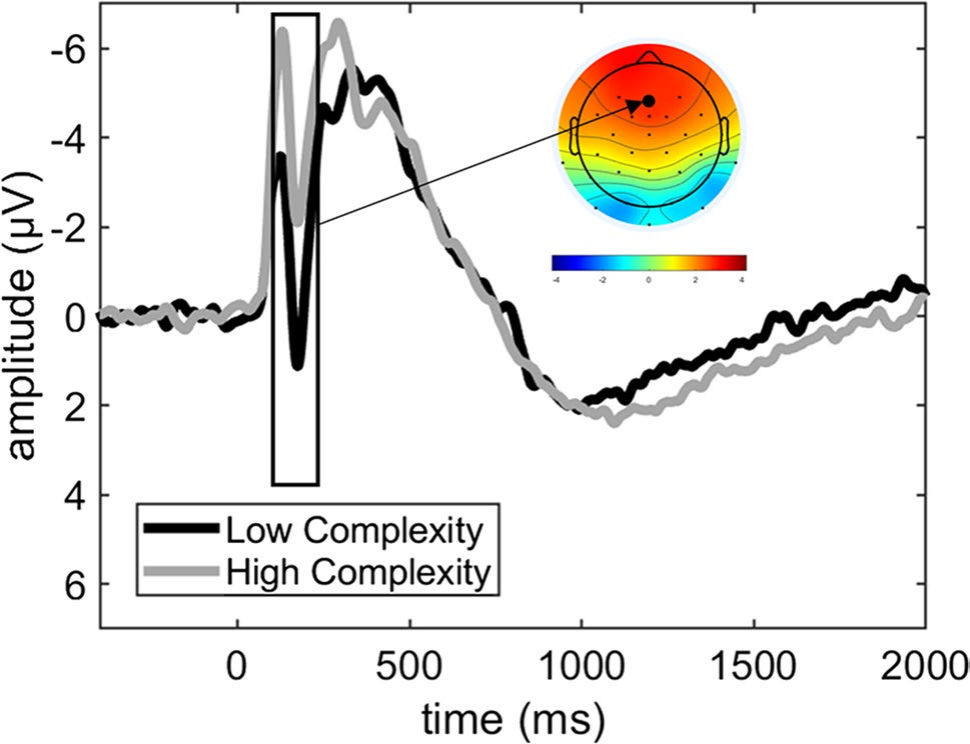
Early frontal negativity at electrode Fz during encoding. The main effect for stimulus complexity is illustrated, averaged across study ratings and first vs. second stimuli of a pair. The box marks the time window of significant differences; the scalp map shows the distribution of the difference between conditions (LC-HC)

The mass univariate ANOVA of the early frontal components included electrodes F7, F3, Fz, F4, F8, FC5, FC1, FCz, FC2, and FC6 and a time window of 100–400 ms. There was a main effect of stimulus (first vs. second stimulus of a pair) in a cluster containing all electrodes and spanning the entire duration of 100–400 ms. More negative-going amplitudes were elicited by the second, compared to the first, stimulus. Furthermore, there was a main effect for condition (HC vs. LC) in a cluster that included all electrodes and spanned a time window of 100–210 ms. More negative-going amplitudes were elicited by the HC compared to the LC condition (Fig. 3). There was no effect of imageability (high vs. low) and no interactions.

The N400 region of interest (ROI) included electrodes P7, P3, Pz, P4, P8, CP1 and CP2, C3, Cz and C4 as well as a time window of 300–500 ms. There was a main effect of stimulus in a cluster containing electrodes Pz, P8, CP1 and CP2, C3, and Cz and spanning the entire time window. More negative-going amplitudes were elicited by the second, compared to the first, stimulus of a pair. Furthermore, there was a rating × stimulus interaction in a cluster containing all electrodes and a time window of 420–480 ms. For the second stimulus only, ERP amplitudes were more negative-going for trials in which the interaction was rated as difficult to imagine. There were no main or interaction effects involving the factor condition.

The frontal slow-wave ROI included a time window of 800–2,000 ms and electrodes F7, F3, Fz, F4, F8, FC5, FC1, FCz, FC2, and FC6. A main effect for stimulus was observed in a cluster that included all electrodes and time points. In line with Kamp et al. (2022), where we already reported this effect for the LC condition, more positive-going slow waves were observed for the first compared to the second stimulus. Furthermore, the stimulus by rating interaction was significant in a cluster containing all electrodes, with a right frontal maximum, and a time window of 800–1,350 ms. Pairs with a high imageability rating elicited a more positive-going slow wave only for the second stimulus of a pair. There were no main or interaction effects involving the factor condition.

#### ERPs during item recognition

The analysis of the mid-frontal old/new effect during item recognition included a time window of 300–500 ms and eight fronto-central electrodes (F3, Fz, F4, FC5, FC1, FCz, FC2, FC6). There was a spatio-temporal cluster with a significant old/new effect spanning a time window of 350–470 ms that included electrodes F3, Fz, F4, FC5, FC1, FCz, and FC2, which was maximal at FCz. There was no interaction.

**Fig. 4:**
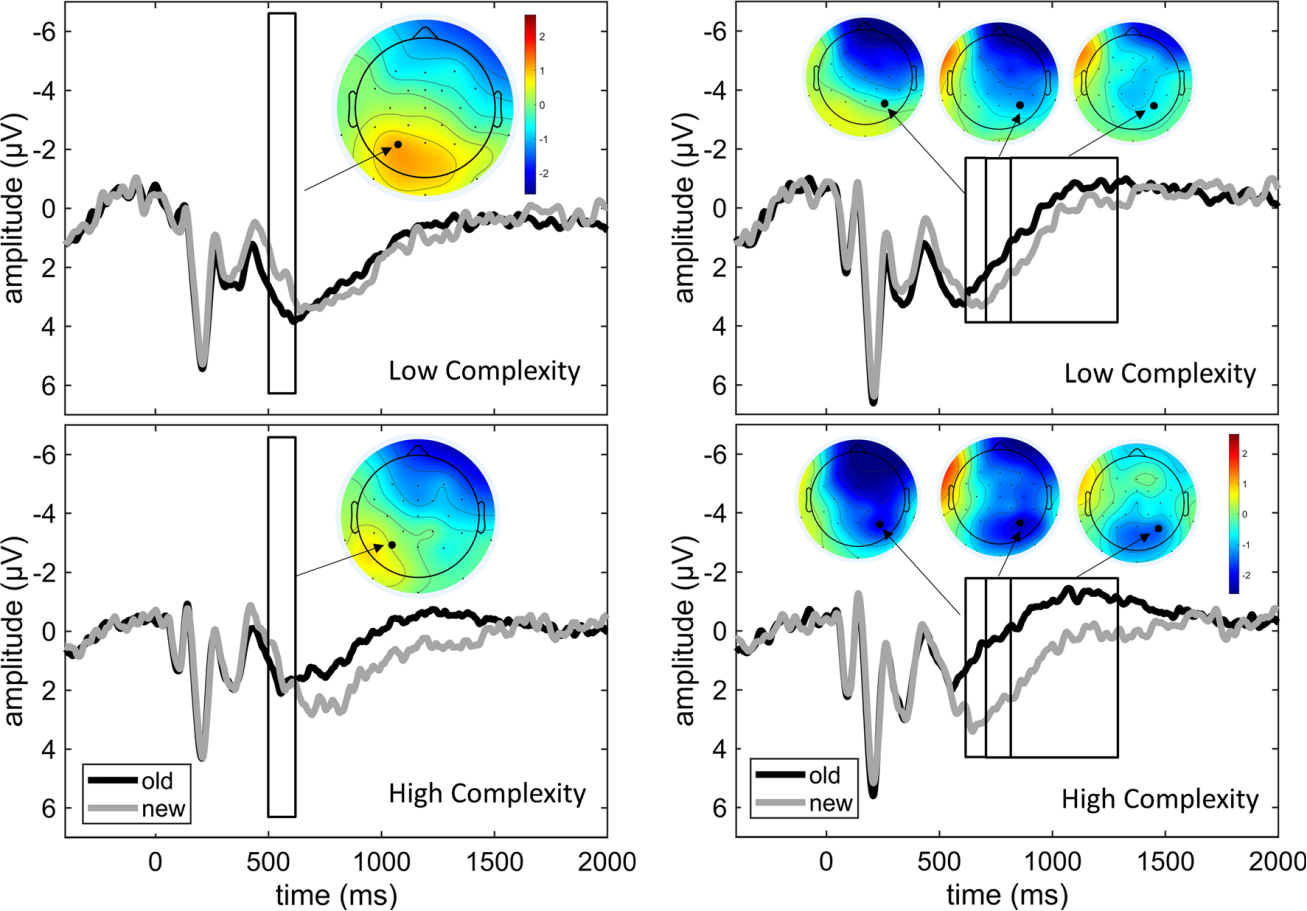
Left parietal old/new effect and LPN. Grand average ERPs elicited by old and new items in the LC (upper panels) and HC (lower panels) conditions during item recognition are shown for the left parietal electrode P3 (left) and the right parietal electrode P4 (right). Left: The time window where the left parietal old/new effect was significant is marked by the box; the scalp distribution of the old/new difference (old-new) is illustrated in the topographic plot. Right: In both the first and the second time window, which were part of the analysis of the left parietal ONE and the LPN, respectively, a negative-going ONE was observed, which was larger in the HC than in the LC condition. The third box marks a time window in which the ONE was significant, but no interaction occurred with condition (although numerically, the ONE remained larger in the HC condition)

The analysis of the left parietal old/new effect during item recognition included a time window of 500–700 ms and seven centro-parietal electrodes (P7, P3, Pz, P4, P8, CP1, CP2). Two spatio-temporal clusters showing significant old/new effects were found. The first cluster spanned a time window of 500–620 ms, included electrodes P3, P7, CP1, Pz, and CP2, and was of positive polarity (i.e., old items elicited a larger amplitude than new items). Hence, it represented the left-parietal old/new effect (Fig. 4, left panel). The second cluster spanned 610–700 ms and included electrodes CP1, P3, Pz, CP2, P4, and P8. This effect showed a reverse polarity. Furthermore, in the analysis of the condition × old/new interaction, there was a significant spatiotemporal cluster spanning 570–700 ms that included electrodes P3, Pz, P4, and P8 (Fig. 4, right panel). This indicates that the old/new effect of reverse polarity was larger for the HC than for the LC condition.

The analysis of the LPN during item recognition included a time window of 700–1,500 ms and parietal and posterior electrodes P7, P3, Pz, P4, P8, O1, O2, and Iz. A significant old/new effect was found in a spatiotemporal cluster including all electrodes and spanning a time window of 700–1,350 ms. This effect represented the LPN and appeared to be a continuation of the reverse old/new effect in the previous time window. There was also a spatiotemporal cluster with a significant condition × old/new interaction, spanning a time window of 700–880 ms and including electrodes O1, O2, and Iz. Hence, this analysis (consistent with the previous time window) suggested that the LPN was larger in the HC condition (Fig. 4, right panel).

### Summary and interim discussion

The only overall difference in ERP amplitudes between the conditions during encoding was an enhanced frontal N2 in the HC condition. This demonstrates that our manipulation of visual complexity affected stimulus processing at early, low-level attentional stages, consistently with prior studies (Bradley et al., 2007; Shigeto et al., 2011). Pairs that were rated as easy to imagine elicited a reduced N400 and an enhanced frontal slow wave to the second stimulus of a pair. This suggests that interactions that were easier to imagine were characterized by facilitated semantic access and enhanced elaboration for the second stimulus. Although we did not find any condition differences in these processes, it must be noted that due to low trial numbers in the “subsequent miss” category, we were unable to conduct a subsequent memory analysis, which could have revealed a stronger relevance for semantic assess and/or elaboration for subsequent memory in one of the conditions (e.g., Mecklinger & Kamp, 2023).

We hypothesized that the late parietal old/new effect during item, reflecting recollection, would be enhanced in the HC condition. However, we found no condition differences in the early mid-frontal or the left parietal old/new effect. The parietal old/new effect is associated with the retrieval of contextual information (Rugg et al., 1996; Wilding & Rugg, 1996). Given that interactive imagery creates strong associative links and is an effective strategy for associative memory (Paivio, 1969), it is possible that in both conditions, associative retrieval was already initiated when the first stimulus of a pair was presented. Alternatively, it is possible that due to the fact that relatively dissimilar items were to be distinguished, recollection of specific details may not have been critical for the recognition decision, thus attenuating differences in recollection between the conditions.

Finally, the LPN during item recognition was larger in the HC than the LC condition, suggesting a higher effort associated with reconstructing the study episodes of complex stimuli. This could pertain to the individual item memory trace or the retrieval of the association, perhaps including imagined details of the interaction, which could have already been initiated upon presentation of the first item (see General discussion). These results suggest that, as far as ERPs during item recognition go, the visual complexity of familiar objects modulates retrieval processes primarily by influencing the effort engaged in reconstructing item and/or associative information regarding the study episode.

The enhancement in early attentional/perceptual processes during encoding, and in the reconstruction of the more complex study episodes during item recognition, was accompanied by an enhanced item memory performance for the colored naturalistic objects. The present manipulation of stimulus complexity presumably led to memory traces that contained more distinctive features that were diagnostic in the distinction between old and new objects. Stimulus complexity did not have a significant effect on associative memory, in line with Bender et al.’s (2017) results for young adults.

One limitation of Experiment 1 is that we tested associative memory only for trials with item recognition hits (as well as item false alarms, for which associative recognition was, however, not analyzed). In principle, a correct associative recognition judgment may be provided even when the item memory judgment regarding the individual objects incorrect. Hence, in Experiment 2 we modified the contingency of the associative memory test on correct item memory. We discuss potential consequences of the difference in the testing procedure in the General discussion.

## Experiment 2

In Experiment 1, although early visual ERP components suggested that complex images drew enhanced attentional resources at an early processing stage during encoding, based on the descriptive trend in the opposite direction, a trade-off between item and associative memory due to the present stimulus complexity manipulation can tentatively be rejected for young adults. However, a different result pattern may emerge in populations with a more vulnerable associative memory like older adults, who typically show much stronger reductions in associative than item memory (Naveh-Benjamin, 2000).

The mechanism underlying the age-related associative memory deficit are to date not fully understood. One under-studied contributor could be an age-related perceptual decline, which also affects color perception (Gulya et al., 2002; Park & Puglisi, 1985), and which generally correlates with lower memory performance (Zheng et al., 2018). Thus, perceptual decline may lead to an increased effort in encoding complex items, leaving insufficient resources for associative encoding in older adults. In light of generally reduced processing capacities, stimulus complexity may hence lead to a trade-off between item and associative encoding in older adults, thus enhancing the age-related associative deficit.

Alternatively, a naturalistic color photograph of a familiar object may make the item more realistic and easier to identify (Brodeur et al., 2017; Faubert, 2002; Wichmann et al., 2002). Hence, when stimulus complexity is manipulated through color photographs of familiar objects, the object may be processed more deeply, and more varied cues may be generated that potentially aid the formation of an association in older adults. This may in turn help older adults alleviate their associative deficit.

### Methods

#### Participants

To detect an effect size of f = .26 with a power of 1- β = 0.95 and α = 0.05 for a three-way interaction between condition (HC vs. LC), group (young vs. old), and test type (item vs. associative) in a mixed factors ANOVA, a sample of N = 158 is required (Faul et al., 2007). Eighty young and 80 older subjects were randomly assigned to the HC condition or the LC condition. Data from one older subject from the LC condition was excluded due to inadequate completion of the experimental procedure as evidenced by a break of about 30 min between study and test, and one younger subject from the HC condition was excluded due to chance performance in item recognition. This resulted in a final sample of 79 older (46 females, 33 males; 60–83 years old, M = 69.10 years, SD = 5.96) and 79 younger participants (45 females, 34 males; 18–35 years old, M = 23.33 years, SD = 3.91).

The older participants were drawn from an existing database. For each older adult that participated, we donated 5€ to a charity. The young adults were recruited through a participation platform and by social media and received credit points. Participants who had taken part in our previous online study (Endemann & Kamp, 2022) or in Experiment 1 were excluded.

The older adults completed the Telephone Interview for Cognitive Status TICS (Brandt, Spencer & Folstein, 1988). None of the participants exhibited signs of cognitive abnormalities (all TICS scores >26), and the TICS did not differ between the LC (M = 39.60, SD = 3.90) and the HC condition (M = 38.6, SD = 3.80; *t*(78) = 1.43, *p* = .26).

#### Procedure and task

The young adults accessed the task via the participation forum. Older participants completed the TICS in an individual phone appointment and were subsequently sent the link to the task via email. The task was administered using E-prime Go 1.0 software.

The encoding phase was identical to Experiment 1. The break between encoding and retrieval was self-paced and did not include any questionnaires. Regarding the test phase, a notable change concerned the association memory test. In Experiment 1, associative memory was tested only for trials with item recognition hits. However, associative memory can in principle be successful even when item memory is unsuccessful. Hence, in Experiment 2, the associative memory test was conducted for old items regardless of the correctness of the item recognition response. This modification allowed us to explore whether the results of Experiment 1 were due to specifics of the memory task (i.e., the associative memory measure being contingent on successful item memory).^1^ However, the main analyses in the present paper are kept equivalent to Experiment 1 for the purpose of comparability between experiments.

#### Analysis of behavioral data

For the encoding phase, we analyzed the proportions of “well” judgments in a 2 (condition: LC vs. HC) × 2 (group: young adults vs. older adults) ANOVA. For the Pr-scores, a mixed ANOVA was calculated with the between-subject factors group (old vs. young) and condition (LC vs. HC) and the within-subject factor test type (item vs. associative). For model comparison we conducted Bayesian mixed ANOVAs analogously to Experiment 1.

### Results

A 2 (condition) × 2 (group) ANOVA on the encoding ratings revealed no main or interaction effects (all *p*-values > .05; Fig. 2; young adults: LC: M = .57; SD = .15; HC: M = .58; SD = .18; older adults, LC: M = .52; SD = .18; HC: M = .57; SD = .17). This was confirmed in the Bayesian analysis, which revealed that the null model best explained the encoding rating data. The second-best model included a main effect of group, but this model was less than a third as likely as the null model, *BF*_10_ = .28. There was hence moderate evidence against main effects of condition, group, or an interaction.

The 2 (group) × 2 (condition) × 2 (test type) ANOVA on the Pr-scores revealed main effects for test type, *F*(1, 154) = 192.14, *p* < .001, η_p_^2^ = .55, and group, *F*(1, 154) = 14.68, *p* < .001, η_p_^2^ = .09, qualified by a test type × group interaction,* F*(1, 154) = 67.68, *p* < .001, η_p_^2^ = .31. Older adults showed lower associative, *t*(156) = 5.74, *p* < .001, but not item memory, *t*(156) = -0.85, *p* = .40, than young adults. The main effect for condition also reached significance, *F*(1, 154) = 14.28, *p* < .001, η_p_^2^ = .09: Overall, memory performance was better in the HC (M = .78; SD = .14) than in the LC condition (M = .70; SD = .14). No other interactions were significant (all *p*-values > .05) (Fig. 2; Table 1).

The Bayesian ANOVA confirmed that a model containing three main effects and a test type × group interaction explained the data best. This model was nearly twice as likely compared to the second-best model, which additionally contained a condition × group interaction, *BF*_*10*_ = .44. The third best model contained all three main effects, a test type × condition and a test type × group interaction. Compared to the best model, the model was a fifth as likely, *BF*_*10*_ = .21. Hence, the Bayesian ANOVA provided evidence against a three-way interaction. Therefore, the stimulus complexity manipulation did not affect the magnitude of the age-related associative memory deficit.

#### Comparison of Experiments 1 and 2 in young adults

In Experiment 1, associative memory was unaffected by stimulus complexity, while in Experiment 2, high complexity was associated with similar advantages in both item and associative memory in both age groups. This difference did not appear to be due to a lower statistical power in Experiment 1, because the test type × condition interaction was significant, and a Bayesian analysis revealed evidence against models that did not include the interaction. Furthermore, the difference cannot be easily explained by changes to the associative memory task, because the analyzed associative memory measure was equivalent between experiments.

We conducted an exploratory analysis to directly compare the experiments for the young adults. A 2 (condition) × 2 (experiment) ANOVA on the encoding ratings revealed a non-significant trend for a main effect for experiment, *F*(1, 121) = 3.25, *p* = .07, η_p_^2^ = .03, and no significant condition × experiment interaction, *F*(1, 122) = 1.10, *p* = .30, η_p_^2^ = .01. The Bayesian ANOVA revealed that the null model was the best fitting model, closely followed by the model including a main effect of experiment, *BF*_*10*_ = .85. The third-best model, including a main effect of condition, was only about a fifth as likely, BF_10_ = .22. Hence, there was inconclusive evidence regarding the possibility that encoding ratings were overall lower in Experiment 2 than in Experiment 1, but moderate evidence against a main effect of condition or an interaction.

A 2 (experiment) × 2 (condition) × 2 (test type) ANOVA on the Pr-scores revealed a main effect for condition, *F*(1, 121) = 13.23, *p* < .001, η_p_^2^ = .10, and a main effect for experiment, *F*(1, 121) = 4.98, *p* = .003, η_p_^2^ = .04, qualified by an experiment × test type interaction, *F*(1, 121) = 27.70, *p* < .001, η_p_^2^ = .17. There was no significant difference between experiments in item memory, but associative Pr-scores were significantly higher for Experiment 1 than for Experiment 2 (Table 1, Fig. 2). Furthermore, the interaction test type × experiment, *F*(1, 121) = 4.74, *p* = .03, η_p_^2^ = .04, and the three-way interaction, *F*(1, 121) = 7.50, *p* = .007, η_p_^2^ = .06, reached significance, reflecting that the difference in associative memory between experiments was larger for the LC (M = .15) than for the HC (M = .05) condition.

In the Bayesian analysis, the best-fitting model included all three main effects, a test type × condition, a test type × experiment, and the triple test type × condition × experiment interaction. The second-best model included all three main effects and the test type × experiment interaction, *BF*_*10*_ = .62. The third best model included the three main effects and the test type × experiment interaction as well as the test type × condition interaction, *BF*_*10*_ = .41. All other models were less than a third as likely as the best model (*BF*_10_ < .32). Taken together, there was moderate evidence for a test type × experiment interaction, suggesting that associative memory was selectively reduced in Experiment 2 compared to Experiment 1. Evidence regarding the three-way interaction and hence regarding a potentially stronger effect of stimulus complexity on associative memory (relative to item memory) in Experiment 1 was inconclusive.

## General discussion

The present study investigated how visual complexity affects memory encoding and retrieval processes as well as item and associative memory performance. We demonstrated that both young and older adults showed better item memory performance for naturalistic color photographs of objects (high complexity) than for monochrome, schematic pictures (low complexity). Associative memory was statistically unaffected by the complexity manipulation in Experiment 1, but was better in the HC than in the LC condition in Experiment. In Experiment 1, an enhanced frontal N2 during encoding suggested that the complex objects were associated with enhanced early attentive processing. Furthermore, an enhanced LPN during item recognition indicated that memory traces of the complex stimuli were associated with a greater effort in reconstructing the learning episode. In Experiment 2, we replicated the relative associative memory deficit of older adults in both conditions. The complexity of the stimulus material did not modulate the magnitude of the relative deficit.

### Effect of stimulus complexity on neurocognitive (ERP) memory processes

The enhanced frontal N2 elicited by the complex stimuli during encoding is consistent with ERP effects that have been previously reported for manipulations of image complexity (Bradley et al., 2007; Folstein & Van Petten, 2008; Shigeto et al., 2011). This pattern supports the idea that early attentive processes are enhanced during memory encoding when the stimuli are visually more complex.

We did not find any evidence for effects of stimulus complexity on higher-level processes like semantic access (N400) or elaborative encoding (frontal slow wave), although both varied with the participant’s own judgment on how well an interaction could be imagined. It is, however, premature to conclude that the conditions did not differ in the extent to which these processes contributed to memory encoding. Specifically, subsequent memory effects (SMEs), denoting differences in activity elicited at encoding depending on subsequent memory retrieval success, are often independent of main condition effects, and are better indicators of which neurocognitive processes are relevant for subsequent retrieval (Mecklinger & Kamp, 2023). In Experiment 1, we were unable to analyze SMEs, because item memory performance was too high in the HC condition, leaving too few trials in the “subsequent miss” category for a sufficient signal-to-noise ratio. We can thus conclude that, as a whole, elaboration and semantic access did not differ between the two conditions, but whether the relevance of these processes to successful encoding differs by stimulus complexity remains elusive.

An increased effort for reconstructing the study episode, reflected in an increased amplitude of the LPN in the HC condition, is consistent with the view that complex stimuli elicit higher-order cognitive processes during retrieval (Hease & Czernochowski, 2015; Sommer et al., 2018). Successful encoding of the varied features of the naturalistic representation of the objects (such as multiple colors vs. black color only) may thus be used as retrieval cues to reconstruct the episode at higher resource demand and task difficulty. Our LPN findings are thus generally in line with Cycowicz (2001), who presented subjects with simple line drawings in red or green. In the memory test, the subjects were either asked to indicate whether they had previously seen the item in one of the colors or whether it was a new item, or to make only an old/new decision. In both conditions, a parietal old/new effect was found, but only in the condition with the color cue did an LPN occur. Thus, the LPN is enhanced when the demands on reconstructing details of the study episode are high (Mecklinger et al., 2016). However, given the nature of our task, it is unclear whether this pertained to the features of the individual item memory trace, or whether the enhanced LPN in the complex condition reflected effortful reconstruction of the associative memory trace, for example including the detailed retrieval of the interactive image. In a two-step recognition task containing a yes/no judgment and subsequently a remember/know judgment, Leynes and Phillips (2008) showed that the LPN was already elicited by the yes/no probe, and it emerged earlier for subsequently “remember” judgments. An LPN for the item-recognition test of our experiment could hence also reflect retrieval of contextual details in preparation for the subsequent associative recognition decision. To differentiate between these possibilities, future research should employ completely separate item and associative recognition tests rather than a single task with a two-step test approach.

#### Effect of stimulus complexity on item versus associative memory

The increased early perceptual and attentional processes during encoding, and the more effortful reconstruction of the study episode during retrieval for the complex, naturalistic images was associated with an advantage in item memory across both experiments and age groups. However, this memory enhancement did not occur at a cost to associative memory, as associative memory was statistically unaffected by complexity (Experiment 1) or enhanced (Experiment 2) in the HC condition. Although other studies have shown that a stronger attentional focus on item encoding may occur at the expense of associative memory (Forester & Kamp, 2023; Hockley & Cristi, 1996; Kamp, 2020), it is important to note that in the present study the complex stimuli also exhibited a more realistic presentation. Color as a property can thus support perceptual processing by making the item more distinguishable, natural, and therefore easier to identify (Brodeur et al., 2017; Faubert, 2002; Wichmann et al., 2002). This, in turn, could have facilitated semantic and/or elaborative processing (although this idea is not directly supported by encoding ERPs due to the lack of SME analyses), thereby supporting the formation of associative traces. Whether an enhanced item-encoding focus (in this case due to perceptual complexity) occurs at the expense of associative memory, or whether it scaffolds associative memory, thus may depend on the nature of the item-encoding processes (especially purely perceptual vs. semantic) that specifically support memory encoding and retrieval (Forester & Kamp, 2023).

Although our results clearly speak against a tradeoff between item and associative memory due to stimulus complexity, our findings regarding associative memory were not unambiguous: In experiment 2, but not in experiment 1, associative memory was higher in the HC compared to the LC condition in young adults.

There was a tendency for higher ratings of successful interactive imagery in Experiment 1 compared to Experiment 2, which could indicate a more effective use of the interactive imagery strategy and hence a deeper processing of the association. This is in line with the overall higher associative memory performance in Experiment 1, while the experiments did not differ in item memory. A potential reason for a less effective strategy use in Experiment 2 could be that due to the online format, participants were distracted by environmental factors, essentially leading to divided attention. Although previous studies have suggested that divided attention leads to equivalent reductions in item and associative memory performance (Kilb & Naveh-Benjamin, 2007; Naveh-Benjamin et al., 1998; Naveh-Benjamin et al., 2003), unlike most prior studies, which used a well-controlled dual-task setting, we had no control over the nature of the (environmental) distraction. Relatively effortless secondary tasks, such as listening to music, could thus have a milder effect of divided attention, which in turn could have more strongly affected associative than item memory, because the former is generally more vulnerable.

Another factor that differed between Experiments 1 and 2 was a difference in the contingency of the associative memory test on item memory. In Experiment 1, an associative test was conducted only for item hits and for item false alarms (i.e., whenever the item was judged “old”). On average, this resulted in 108.6 (LC) and 110.4 (HC) association test trials in Experiment 1, of which some (10.8 or 4.8 in the LC and HC condition, respectively) were composed of two completely new items. By contrast, in Experiment 2, associative memory was tested in all old trials (but not after item tests of new items), resulting in 120 associative memory test trials, which were all composed of old items (either as old or a recombined pairs). Hence, the number of trials in which an associative test was conducted, as well as the nature of some of the associative memory tests, differed slightly. Output interference during recognition testing (Aue et al., 2015; Criss et al., 2011; Wilson et al., 2020), although its effects are not well studied in associative memory, could thus have had a stronger effect in Experiment 2, leading to a reduced associative memory performance. However, this potential difference in output interference between experiments was largely similar for both conditions.

In sum, the very high associative memory performance in Experiment 1 could have masked a potential facilitation of associative memory through the stimulus complexity manipulation. Although we cannot completely resolve the issues raised here, overall, we consider it likely that with the present experimental manipulation, when (associative) memory performance is sufficiently low both item and associative memory can benefit from enhanced complexity.

#### No effect of stimulus complexity on the age-related associative memory deficit

In Experiment 2, we demonstrated that the age-related associative deficit was independent of stimulus complexity: Both older and younger adults showed enhanced item and associative memory performance in the HC compared to the LC condition. These results differ from Bender et al. (2017), who reported that visual complexity led to a trade-off between item versus associative memory in older adults, effectively amplifying their associative memory deficit. As outlined in the Introduction, Bender et al.’s complex images contained background and contextual information and may have thus required stronger inter-item binding processes within each image. In our study, by contrast, visual complexity pertained only to the object itself, which was potentially a cleaner manipulation of item complexity. Another difference is that we used pairs of familiar objects, while Bender et al. (2017) used faces in combination with a first name and a last name. Faces and objects are processed through different mechanisms (Maurer et al., 2002; Tanaka & Sengco, 1997). Hence, the effect of stimulus complexity on associative memory could depend on the type of material and the composition of the associations. Further, since the faces and names were pre-experimentally unfamiliar, the complexity manipulation of Bender et al. (2017) may thus not have affected semantic processing. By contrast, in our study, a facilitation of semantic processes due to the naturalistic presentation may have outweighed the additional perceptual demands, thus scaffolding associative memory rather than drawing resources away from it. Indeed, prior research has shown that older adults benefit from situations in which they can rely on semantic processing (Kamp et al., 2018; Troyer et al., 2006). This may be a reason why their item and associative memory benefitted from the naturalistic stimulus presentation to a comparable extent as for the young adults in the present study. In conclusion, stimulus complexity may support associative memory in young and older adults when it also facilitates semantic processes.

#### Limitations, conclusion, and future directions

The present results are limited by the use of a between-subjects design, which was necessary in Experiment 1 to have enough trials for ERP analysis while the experiment retained a feasible duration. Furthermore, since we used completely different images in the two conditions, although they depicted the same kinds of objects, we cannot be certain that the stimulus set was equated on all aspects other than visual complexity, which are relevant to memory.

In conclusion, we found that complex naturalistic object presentations, which attracted early attentional processing during encoding and elicited a higher effort to reconstruct item and/or associative information from the study episode (Experiment 1), improved item memory (Experiments 1 and 2), and left associative memory unaffected (Experiment 1) or increased associative memory (Experiment 2), compared to less complex objects. These memory advantages were found in both young and older adults and may have been due to semantic processes being facilitated by the naturalistic stimuli. When a higher load of perceptual analysis is accompanied by a facilitation in semantic processing, stimulus complexity can scaffold associative memory processes rather than drawing resources away from it.

Future research is necessary to examine whether older adults, like the young adults in Experiment 1, show enhanced LPN during retrieval of complex stimuli, which would demonstrate that complexity affects memory in both age groups via similar mechanisms. In addition, future research should test whether similar effects of visual complexity on item and associative memory would be observed with a task instruction that facilitates associative binding less strongly than interactive imagery.

## Data Availability

The behavioral data are available at https://osf.io/t7ayv/?view_only=af0365fb0ae1485bb535767deff0ab0f. The EEG data are available upon request to the authors.

## Appendix: English object labels referring to the images used in the experimental task

### A. Objects included in both conditions (HC and LC)

Acorn, Airplane, Anchor, Angel, Ant, Antenna, Apple, Arch, Baby, Backpack, Bandaid, Bag, Ballerina, Balloon, Banknotes, Bathrobe, Bathtub, Beaver, Bed, Beetle, Bell, Belt, Bench, Bicycle, Bird, Book 1, Book 2, Boot, Bottle, Box, Bra, Bride, Broom, Brush, Bucket, Buffalo, Bus, Butterfly, Cactus, Cake, Camel, Candy, Candy cane, Car, Carrot, Castle, Cat, Caterpillar, Chainsaw 1, Chainsaw 2, Chair, Chameleon, Cheese, Cherry, Church, Clamp, Cleat, Clip, Clock, Cloverleaf, Cobra, Coffin, Comb, Corkscrew, Corn, Couch, Cow, Crocodile, Croissant, Crow, Crown, Cucumber, Cup 1, Cup 2, Cupboard, Daisy, Deer, Desk, Dice, Dog, Dolphin, Donut, Door handle, Dress, Dresser, Drill, Drum, Duck, Egg, Eggplant, Elephant, Envelope, Fairy, Fan, Faucet, Feather, Film, Fishing rod, Flag, Folder, Frog, Fruit, Ghost, Giraffe, Glass, Glasses, Globe, Golf ball, Grapes, Grasshopper, Guitar, Gymnast, Hairband, Hairpin, Hand, Handbag, Helmet, High chair, Holiday clothing, Hook, Human, Ice, Jacket 1, Jacket 2, Jalapeno, Jam, Jewelery, Jigsaw puzzle, Juice, Kangaroo, Kayak, Kethchup, Key, Kite, Kiwi, Knife, Ladder, Lamb, Lamp, Laptop, Lawnmower, Leaf, Leopard, Life saver, Light bulb, Lime, Lion, Lizard, Lock, Mascara, Matches, Meat, Microscope, Microwave, Monastery, Monkey, Moon, Mosquito, Moths, Mouse, Mousetrap, Nail, Octopus, Oranges, Oven, Owl, Package, Pan, Panda, Pants, Pasta, Pear, Pencil, Penguin, Perfume, Piano, Pig, Pigeon, Pineapple, Piranha, Pitchfork, Pizza, Playing card, Pliers, Popcorn, Printer, Pudding, Pumpkin, Queen, Rabbit, Radio, Radish, Rattle, Record, Referee, Rhinoceros, Rice, Rooster, Rope, Rug, Ruler, Runner, Safety pin, Sailboat, Salad, Salt, Sandpit, Sandwich, Sauce, Sausage, Scarf, Scissors, Sewing machine, Shark, Shell, Shirt, Shoe, Shovel, Skateboard, Skipping rope, Skunk, Sledge, Slide, Snail, Snow, Snowman, Sock, Soup, Spatula, Spoon, Stapler, Stroller, Suitcase, Swing, Syringe, Table tennis racket, Pill, Teapot

Teeth, Telephone, Tent, Tie, Toaster, Toilet, Towel, Tractor, Tree, Trumpet, Tube, Umbrella, Vase, Violin, Waiter, Walnut, Wardrobe, Washing machine, Wasp, Watch, Whale, Wheelbarrow, Whistle, Wild boar, Windmill, Window, Wrench, Zebra.

### B. Objects only included in the LC condition

Airport, Arrow, Bagpipe, Blender, Bone, Bridge, Bulldozer, Cassette, Cello, City, Crane, Donkey, Eel, Fox, Harp, Hut, Lips, llama, Medal, Moose, Needle, Pool, Rails, Roast turkey, Skate, Squirrel, Streetcar, Submarine, Sun, Tire, Tricycle, Truck, Unicycle, Wheelchair.

### C. Objects only included in the HC condition

Basket, Bear, Bicycle, Bow, Bus, Camera, Car, Cassette recorder, Clarinet, Door, Electric guitar, Firetruck, Fist, Fountain, Gazelle, Hedgehog, Horse, House, Ice skate, Inline skate, Lighthouse, Microphone, sMotorcycle, Ostrich, Pillar, Pyramids, Scooter, Shrimp, Skull, Sunflower, Tank, Temple, Toast, Wool.

## Footnotes

1 We repeated the analysis of recognition performance in Experiment 2 including all recognition trials. The main pattern of results was unaffected by this change.
