# Correction: Examining the role of stimulus complexity in item and associative memory

**DOI:** 10.3758/s13421-024-01652-2

**Published:** 2024-10-16

**Authors:** Ricarda Endemann, Siri‑Maria Kamp

**Affiliations:** Department of Neurocognitive Psychology, Trier University, Johanniterufer 15, 54290 Trier, Germany

**Correction: Memory & Cognition**

10.3758/s13421-024-01590-z

The article** “**Examining the role of stimulus complexity in item and associative memory**”,** written by Ricarda Endemann and Siri-Maria Kamp, was originally published electronically on the publisher’s internet portal on 18 July 2024 without open access. With the author(s)’ decision to opt for open access, the copyright of the article changed on 9 October 2024 to © The Author(s) 2024 and the article is forthwith distributed under a Creative Commons Attribution 4.0 International License, which permits use, sharing, adaptation, distribution and reproduction in any medium or format, as long as you give appropriate credit to the original author(s) and the source, provide a link to the Creative Commons license, and indicate if changes were made. The images or other third party material in this article are included in the article's Creative Commons license, unless indicated otherwise in a credit line to the material. If material is not included in the article's Creative Commons license and your intended use is not permitted by statutory regulation or exceeds the permitted use, you will need to obtain permission directly from the copyright holder. To view a copy of this license, visit http://creativecommons.org/licenses/by/4.0/.

